# Use of FFPE-derived DNA in next generation sequencing: DNA extraction methods

**DOI:** 10.1371/journal.pone.0211400

**Published:** 2019-04-11

**Authors:** Samantha J. McDonough, Aditya Bhagwate, Zhifu Sun, Chen Wang, Michael Zschunke, Joshua A. Gorman, Karla J. Kopp, Julie M. Cunningham

**Affiliations:** 1 Medical Genome Facility, Mayo Clinic, Rochester, Minnesota, United States of America; 2 Department of Health Sciences Research, Division of Biomedical Statistics and Informatics, Mayo Clinic, Rochester, Minnesota, United States of America; 3 Pathology Research Core, Mayo Clinic, Rochester, Minnesota, United States of America; 4 Department of Laboratory Medicine & Pathology, Mayo Clinic, Rochester, Minnesota, United States of America; University of Helsinki, FINLAND

## Abstract

Archival tissues represent a rich resource for clinical genomic studies, particularly when coupled with comprehensive medical records. Use of these in next generation sequencing (NGS) is a priority. Nine formalin-fixed paraffin-embedded (FFPE) DNA extraction methods were evaluated using twelve FFPE samples of varying tissue types. Quality assessment included total yield, percent dsDNA, fragment analysis and multiplex PCR. After assessment, three tissue types from four FFPE DNA methods were selected for NGS downstream evaluation, targeted and whole exome sequencing. In addition, two low input library protocols were evaluated for WES. Analysis revealed average coverage across the target regions for WES was ~20-30X for all four FFPE DNA extraction methods. For the targeted panels, the highest molecular tag coverage was obtained with the Kingfisher FFPE extraction method. The genotype concordance was 99% for the commonly called variant positions between all four extraction methods with the targeted PCR NGS panel and 96% with WES. Assessing quality of extracted DNA aids in selecting the optimal NGS approach, and the choice of both DNA extraction and library preparation approaches can impact the performance of archival tissue in NGS.

## Introduction

Next generation sequencing is rapidly becoming established in the clinic, predominantly in oncology but also as a means of diagnosis in individuals with unresolved medical issues. Archival tissue represents a singularly valuable resource for disease oriented research, particularly when combined with comprehensive medical records such as that of the Mayo Clinic. However, DNA extracted from such samples can vary widely in quality due to age, fixation conditions, DNA-protein crosslinking, and inhibitors, which may impact downstream genomic analyses. Samples are typically obtained in the operating room so how they are handled, as well as time exposed to formalin both contribute to potential DNA damage but are typically outside the control of investigators[1].

With the use of highly sensitive NGS applications it is imperative that the FFPE DNA extractions used in these assays be of the best quality obtainable. Variation in pre-processing may lead to inconsistencies in detection of mutations or viral presence[2, 3] and variation in both quantity and quality of DNA extracted at four commercial laboratories was reported by Arreaza et al[4]. Thus, not only DNA quality, but the methods used to extract DNA contribute to performance in downstream assays.

Approaches for extraction of DNA from FFPE samples have been compared by others[2, 5–10]; one used NGS for downstream analysis for two samples only[7], while Bonnet et al[5] used whole exome sequencing (WES) on 42 samples, comparing three DNA extraction approaches but not comparing the same samples extracted by the three methods. Schweiger et al[11], using a controlled fixation of 72 hours for the FFPE-DNA, noted equivalence of three methods but no data were shown; QIAamp was used for seven FFPE samples and revealed that copy number and mutation analyses were possible. Other NGS studies of FFPE-derived DNA extracted by a single method have been reported[11–18]. Kerick et al varied input for a targeted capture-based from 500ng-1.5μg of FFPE-DNA of one of five patients and showed comparable performance using standard library preparation[17].

Recent developments of FFPE DNA extraction processes include: new deparaffinization solutions, repair strategies, and magnetic bead technology. These improvements along with the need to profile archival tissues led us to evaluate several DNA extraction methods, assess low input library preparations for such samples, and use the preferred methods in whole exome and targeted panel sequencing applications. In addition, we applied DNA quality metrics to assess the degree of fragmentation of FFPE DNA. DNA was extracted from 12 blocks of paraffin-embedded, formalin fixed representing several tissue types using nine commercially available extraction methods, including both manual and automated processes. Nine sequential sections were collected from each block to allow comparison of the DNA extraction methods for each of the 12 tissues. These samples were selected to represent a spectrum of quality, from highly cellular to those with high adipose tissue content that typically yield poorer quality DNA [19]. After DNA quality and quantity were assessed, four of the processes were evaluated for performance in NGS technologies. These included two library preparation protocols for low input DNA whole exome sequencing (WES), as limited DNA yields from FFPE samples are common, and two targeted DNA panels.

## Material and methods

### Samples

Mayo Clinic IRB approved the study, all samples were de-identified. Following this approval, 12 paraffin blocks of formalin fixed tissues were selected from Mayo’s Pathology Research Core laboratory’s Control and Assay Development Paraffin Preserved Tissue archive. Tissues included one each of normal and diseased sections from breast, colon, lung, pancreas, along with two unique normal tonsil tissues and two different sections of brain tissue (brain stem and cerebellum). Tissues were fixed for at least 6 hours before being embedded in paraffin. Nine 10 μM sections were cut sequentially from each block using a standard microtome (Leica Rotary Microtome RM2235, Leica Biosystems, Buffalo Grove, IL) and each section was collected onto a separate slide. For each slide, tissue measuring approximately 10 x 15 mm^2^ was scraped using a scalpel and material was placed into a 1.5 ml microcentrifuge tube for DNA extraction. As the samples included in the study were anonymized, it was not possible to compare with matched frozen tissue samples.

### FFPE DNA extraction

We compared both manual and automated extraction methods available for use with FFPE tissues and evaluated extraction time, throughput and quality. Manual protocols evaluated for the study were KAPA Express Extract kit (KAPA Biosystems, Wilmington, MA, USA), Promega Reliaprep FFPE gDNA Miniprep system (Promega, Fitchburg, WI, USA), QIAGEN QIAampFFPE tissue kit, and QIAGEN GeneRead DNA FFPE kit (QIAGEN, Germantown, MD, USA). Automated extraction methods evaluated for the study were QIAGEN QIAsymphony DNA mini kit (QIAsymphony SP), QIAGEN GeneRead DNA FFPE kit (QIAcube), Maxwell RSC DNA FFPE Kit (Promega Maxwell RSC), PerkinElmer chemagic FFPE DNA kit (chemagic MSM 1; Perkin Elmer, Baesweiler, Germany), and Applied Biosystem’s Duo MagMAX FFPE DNA Isolation Kit (KingFisher Duo; Thermo-Fisher Scientific, Waltham MA, U.S.A., no longer available but replaced by A31881 MagMAX FFPE DNA/RNA Ultra Kit combining two kits with an altered workflow). All extractions were performed using one 10 μM section from each of the 12 tissue blocks. All sections were deparaffinized using QIAGEN’s Deparaffinization Solution following manufacturer’s guidelines, exceptions were QIAamp extraction protocol which used xylene and KAPA Express Extract which contains its own extraction buffer and enzyme. Manual and automated protocols for each method were followed according to manufacturers’ guidelines. The only modification made was to the QIAamp FFPE tissue protocol which used an overnight lysis incubation time of 56°C instead of the suggested 1 hour.

All of the methods used a proteinase (Proteinase K in all but KE-M which uses a thermostable protease (not otherwise specified). Three of the four manual methods used silica based mini-elute columns to bind DNA; KE-M did not include a clean-up step. Both GeneRead methods add a repair step that removes formalin crosslinks and de-aminated cytosines. The automated methods all use magnetic beads to isolate the DNA, with the exception of the automated GeneRead protocol which employs the QIAcube (silica based) for column purification. KE-M offered the shortest workflow compared the others, but its lack of a clean-up step outweighed the rapid protocol. The five automated protocols offered the most ease of use, with TKM-A being the most fully automated protocol with the fewest upfront manual sample preparation steps.

### Quality

DNA quality for each extracted sample was measured by evaluating quantity, purity, amount of double stranded DNA, DNA quality number (DQN) and fragment length. Samples were quantified using Qubit dsDNA BR Assay (ThermoFisher Scientific, Waltham, MA, USA), any measurement below 2.0 ng was verified using the Qubit dsDNA HS Assay. Samples were also quantified using NanoDrop NT-1000 spectrophotometer. dsDNA was calculated using the ratio of Qubit to Nanodrop readings. Fragment length and degradation were assessed using the Advanced Analytical Fragment Analyzer High Sensitivity Large Fragment Analysis kit (Agilent Technologies, Santa Clara, CA, USA) and a multiplex PCR assay (Life Science Innovations, Qualitative Multiplex PCR Assay for Assessing DNA Quality from FFPE Tissues, and Other sources of Damaged DNA Issue 23, SigmaAldrich, St. Louis, MO) which uses amplicon size to determine degradation and fragment size. DQN was calculated from the Advanced Analytical Fragment Analyzer High Sensitivity Large Fragment Analysis kit data using a threshold of 500bp. For final selection of methods for downstream analysis, a t-test was performed to calculate p-value based on yield, percent dsDNA and fragment length of each method.

### Sequencing

FFPE DNA samples and one CEPH control (Centre d’Etude du Polymorphisme Humain, Coreill Institute, Camden, NJ, USA) were subjected to two different low input library preparations for whole exome sequencing (WES), NEBNext Ultra II DNA Library Prep (New England BioLabs Inc. Ipswich, MA, USA) and ThruPLEX DNA-seq Kit (Rubicon Genomics, Ann Arbor, MI, USA). Library preparations were made following manufacturer’s guidelines using an input of 50ng of DNA. Library concentrations were assessed and approximately 500ng of each sample library was enriched using Agilent’s SureSelect XT Target Enrichment System V5+UTR (Agilent, Santa Clara, CA, USA). Libraries were quantified and sequenced 13 per lane, PE 150bp on an Illumina HiSeq 4000 (Illumina, San Diego, CA, USA). To further investigate the influence of DNA FFPE extraction methods on downstream next generation sequencing applications, this same set of 13 samples was prepared following manufacturer’s guidelines using the QIAGEN QIAseq Targeted Human Comprehensive Cancer Panel with an input of 40ng FFPE DNA from each sample. All libraries were sequenced in one lane per panel on an Illumina HiSeq 4000, PE 150bp. One sample was also sequenced using the QIAGEN QIAseq Targeted Human Breast Cancer Panel. This targeted approach was selected as it was amenable for use with fragmented DNA and used molecular barcoding to address duplicate reads that are common in NGS using low input and degraded DNA.

### Analysis

Bioinformatics analysis for the WES was performed using an in-house DNA analysis workflow (GenomeGPS v4.0.1). The reads were first aligned to the GRCh37 build of the human reference genome [20] using Burrows-Wheeler Aligner (BWA-MEM v0.7.10) [21] with minimum seed length, matching score, mismatch penalty, gap open penalty and gap extension penalty parameters set to default BWA-MEM values. After alignment, the reads were re-aligned and re-calibrated using Genome Analysis Toolkit (GATK) [22] Indel Realigner (v3.4–46) to optimize the mapping around indels. Variant calling was then performed on the realigned reads using GATK Haplotype Caller and the called variants were functionally annotated using an in-house developed genomics annotation tool BioR [23]. The QIAseq panel analysis was performed using QIAGEN’s GeneGlobe data analysis portal. The portal performs appropriate read trimming, generating consensus reads using unique molecular indexes (UMIs), and variant calling using QIAGEN’s barcode-aware variant caller “smCounter” [24], followed by variant annotation. We used a cutoff of 5x for concordance analyses. For variant calling comparison between methods, we used genotype concordance as a measure to assess the reproducibility of called variants along with the number of variants detected with each method. Concordance was called between methods when the variant position and the variant genotype were identical between methods.

## Results

### DNA extraction

The methods used to extract DNA from the 12 FFPE samples are shown in Table 1 along with the acronym used for each in this report; modifications were not added to the manufacturers’ protocols with one exception noted above.

**Table 1:** DNA extraction methods.

FFPE DNA Extract Kit	Catalog Number	Process	Abbreviation
KAPA Express Extract Kit	KK7100	Manual	KE-M
Promega Reliaprep FFPE gDNA Miniprep System	A2352	Manual	PR-M
QIAGEN GeneRead DNA FFPE Kit	180134	Manual	QGR-M
QIAamp DNA Mini Kit (Mayo’s Biospecimens Accessioning and Processing Lab protocol)	51304	Manual	QA-M
QIAGEN QIAsymphony DSP DNA Mini Kit	937255	Automated	QS-A
QIAGEN QIAcube GeneRead DNA FFPE Kit	180134	Automated	QGR-A
Promega Maxwell RSC DNA FFPE Kit	AS1450	Automated	PM-A
PerkinElmer Chemagic MSM1 FFPE DNA Kit	CMG-1099	Automated	PEC-A
ThermoFisher KingFisher Duo MagMAX FFPE DNA Isolation Kit (No longer available)	Replacement- A31881 MagMAX FFPE DNA/RNA Ultra Kit	Automated	TKM-A

### DNA quality assessment

**Fig 1:**
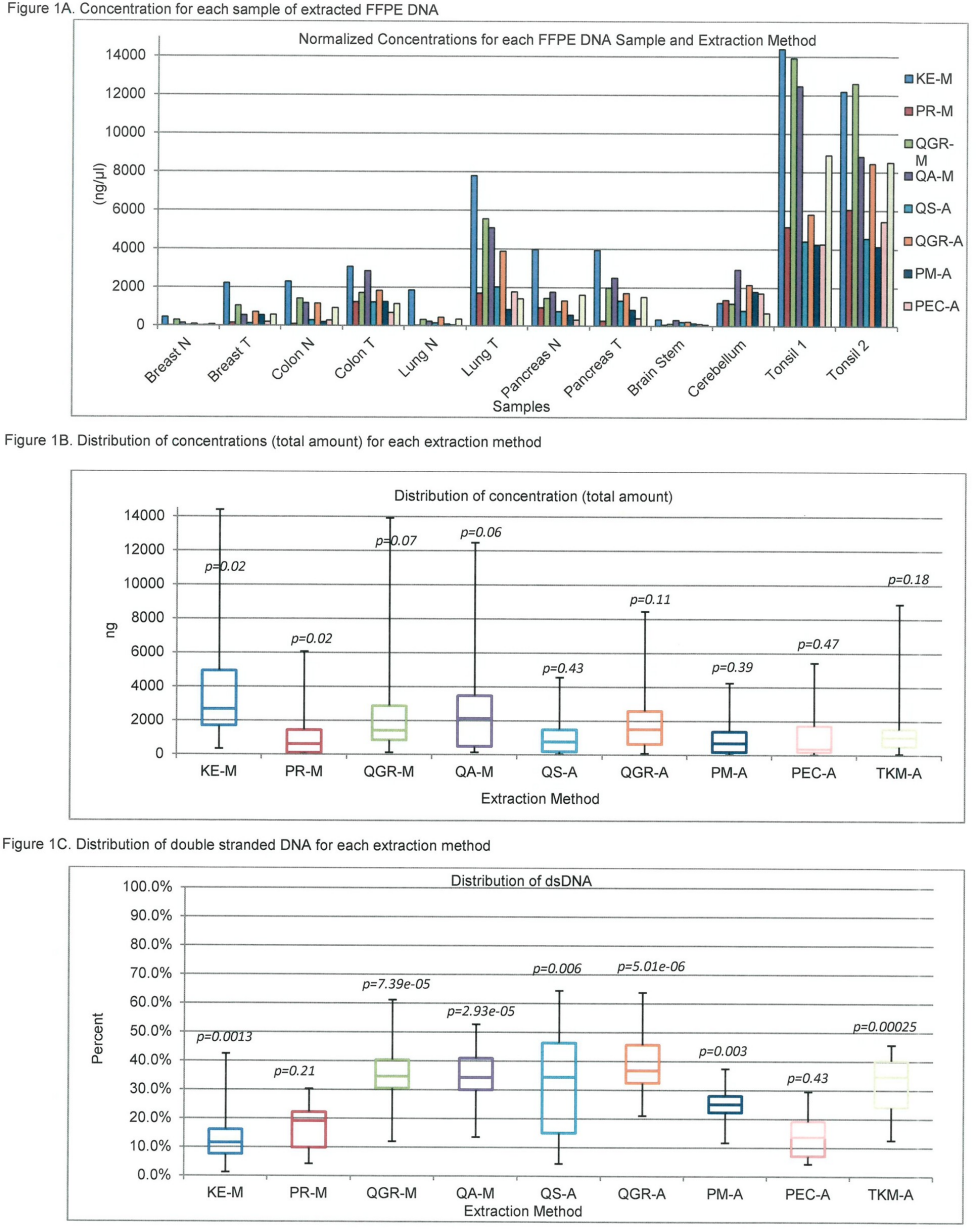
DNA quality metrics. Each extraction method is designated by a specific colour (see key) which is maintained in Fig 2A and 2B. A: Concentration for each sample of extracted FFPE DNA, B: Distribution of concentrations (total amount) for each extraction method, C: Distribution of double stranded DNA for each extraction method.

Data for DNA yield and dsDNA content for the 12 different tissue types used on the four manual and five automated FFPE DNA extraction methods are shown in Fig 1A–1C. Among the tissues, DNA extracted from brain stem and normal breast tissues had the lowest yields of DNA, tumors yielded more DNA than their normal counterparts, and tissue from tonsils yielded the highest (Fig 1A). There was no significant difference between the methods when yield was considered, (Fig 1B). However, when considering percentage of dsDNA, the template required for most downstream assays, QA-M, QGR (both manual and automated), QS-A and TKM-A yielded higher dsDNA percentages (P<0.01, Fig 1C). 260/280 ratios were above 1.8 for all methods except KE-M and PR-M (S1 Fig).

**Fig 2:**
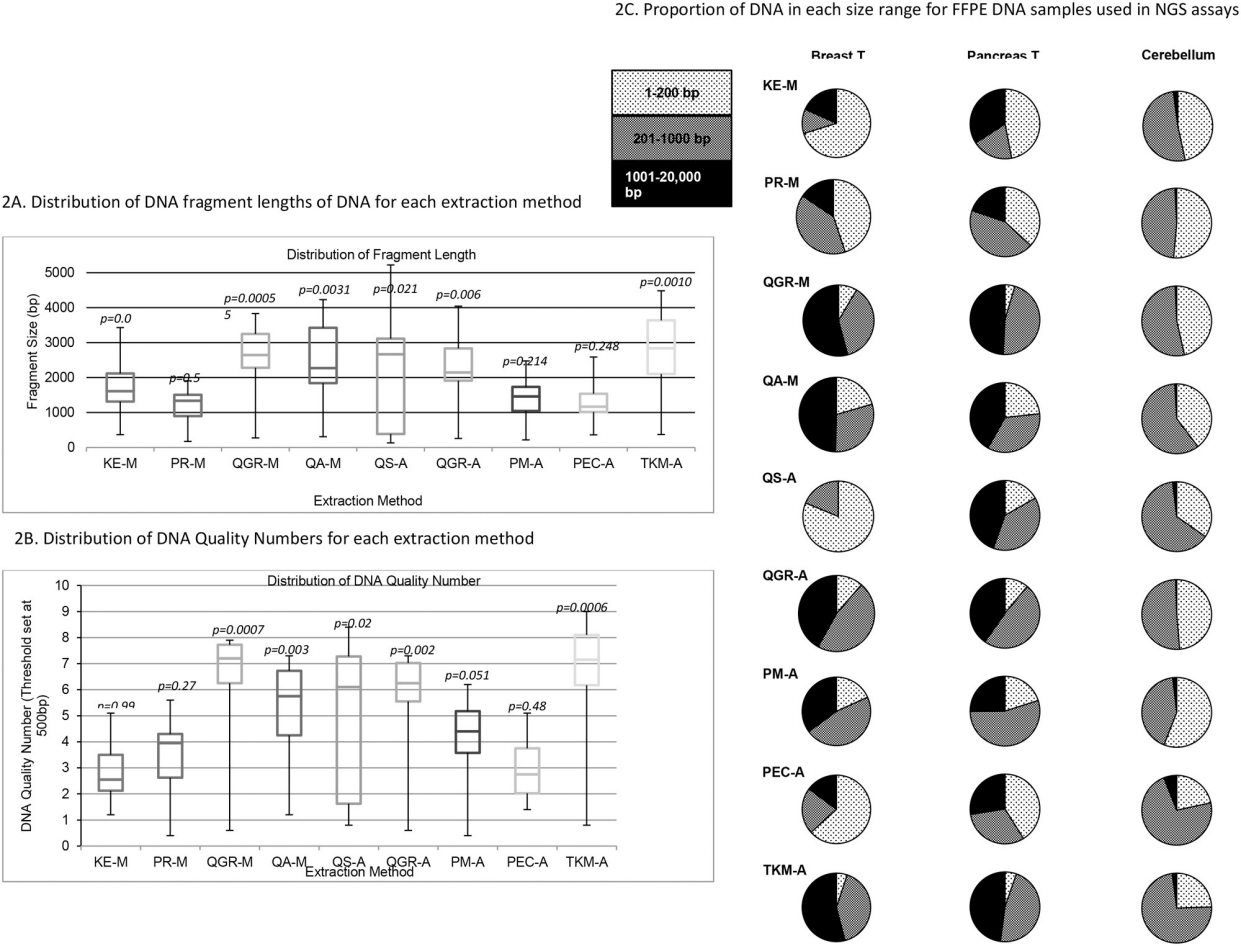
Fragment analysis. A: Distribution of DNA fragment length for each extraction method, B: Median DNA Quality number (DQN) for each method; a size threshold of 500bp was selected for assessment. C: Percent of fragments <200bp, 201–1,000bp, 1,001–20,000bp for three representative tissue types.

Fragment analysis data are shown in Fig 2A–2C with the full data set in S1 Table.

The median fragment lengths of each of the nine approaches are shown in Fig 2A and DQN in Fig 2B. Several methods were poor overall at preserving fragment length, notably KE-M, PR-M, PM-A and PEC-A, while QS-A was more variable. Those that do preserve fragment length most consistently were QGR (manual and automated), QA-M, PM-A and TKM-A methods (P<0.01 for fragment length and DQN). Fig 2C shows the percentage of DNA from three samples (breast tumor, pancreas tumor and cerebellum) of varying quality at 1-200bp, 201-1000pb and 1001–20,000bp for four methods (data for all samples are in S1 Table). More fragmented samples such as brain stem and cerebellum have most DNA <1000bp by most methods. However, for the tumor samples, QGR (manual and automated), QA-M, PM-A and TKM-A methods yielded larger percentages of higher molecular weight DNA.

Fragment analysis gives a sense of the fragmentation of the DNA; however whether it is effective in downstream applications may not follow. Thus, we evaluated performance in a multiplex PCR to determine how well amplicons of different size are amplified compared to a control sample. Data from the multiplex PCR are shown in S2 Table. Results are variable based on method and tissue type. KE-M generally produced poorer results, likely due to the absence of a DNA clean up step. Poorer quality DNA (brain stem, cerebellum) generally produced <20 percent of amplifiable amplicons above 132–196 base pairs with most of the methods. Of all the methods, PEC-A, QS-A, and KE-M produced poor quality results while QGR (both manual and automated) and TKM-A results show consistent amplification of larger amplicons among the samples.

Four DNA extraction methods QGR-M, QA-M, QGR-A and TKM-A yielded a p-value lower than the significance threshold (P < 0.01) for dsDNA, fragment length and DQN and were thus chosen to evaluate in downstream NGS applications. Three of the 12 tissue types evaluated were chosen for this NGS assessment based on their quality scores, ranging from severe to moderate degradation; cerebellum, breast tumor and pancreas tumor.

### Next generation sequencing

**Fig 3:**
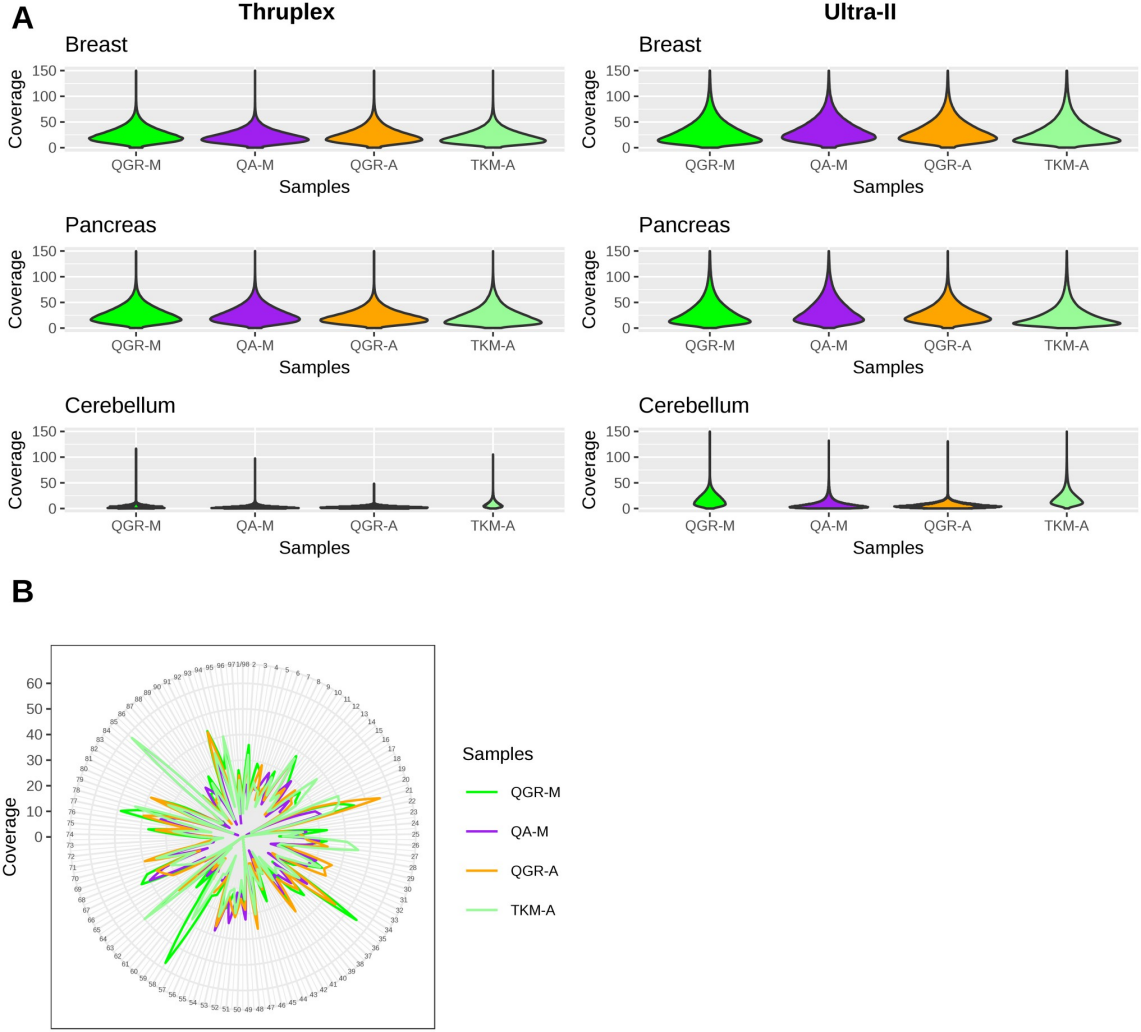
Raw coverage distribution for the four DNA extraction methods for three tissue types. A) Across all targeted bases as seen in the Thruplex (left panel) and Ultra-II (right panel) methods and B) Across a subset of the targeted regions with the Ultra II method and the Breast samples. The numbers 1–98 represent a random set of 98 target regions. The overall coverage is very similar for the four methods. The cerebellum samples show significantly lower coverage compared to other tissues due to the highly degraded nature of the samples. The CIRCOS plot the coverage lines within are noted on the left (0X the inner most to 60X the outer most concentric lines).

For WES, two low input library preparation methods were used along with the four DNA extraction methods; coverage metrics, read duplication rates and fragment insert sizes for each DNA extraction and library preparation method combination are shown in S3 Table. The average value of median coverages from all methods was 23.7X (standard deviation s = 8.61) except for the highly degraded cerebellum samples, with Ultra II providing a slightly higher coverage of 25.88X (standard deviation s = 3.05) than ThruPlex which provided a coverage of 21.5X (standard deviation s = 2.94). Additionally, the distribution of coverage across the targeted bases as shown in Fig 3A and S2 Fig. further suggests that the UltraII method is able to cover some targeted bases even at 75X, while the Thruplex coverage distribution generally tapers off beyond 50X. A circos plot of raw coverage between methods for a subset of exons targeted is shown in Fig 3B.

While we did not observe large differences in read duplication rates between the four DNA extraction methods (Δ_duplication_ = 3.05 percent), we did note that the read duplication rates were lower with the Ultra II library preparation method (11.9 percent) than ThruPlex (26.21 percent). Also, for the most degraded sample, cerebellum, duplication rates were generally higher than for the other tissues types (S3 Table).

Table 2 shows the number of variants called with each method, and the genotype concordance between each pair of methods. For calculating genotype concordance between any pair of methods, only the commonly called variants by the pair were considered. An average genotype concordance of 96% for called variants was observed with a standard deviation of s = 0.57 between the methods, and alternate allele frequencies showed an average Pearson’s Score correlation of r^2^ 0.95.

**Table 2:** Number of variants called (A) and percent genotype concordance (B).

**Tissue/Method**	**QA-M**	**QGR-M**	**QGR-A**	**TKM-A**
**Number of variants called**				
**Breast**	69,516/70,326*	70,463/70,523	69,952/71,249	70,013/72,112
**Pancreas**	69,547/69,488	70,462/70,951	69,468/70,791	71,235/70,968
**Cerebellum**	47,097/61,337	55,316/71,807	50,478/59,878	76,539/83,721
**Percent genotype concordance**				
	**QA-M**	**QGR-M**	**QGR-A**	**TKM-A**
**QA-M**	100/100*	95.56/96.7	95.6/96.72	95.43/96.62
**QGR-M**	95.56/96.7	100/100	95.77/96.59	95.45/95.41
**QGR-A**	95.6/96.72	100/100	100/100	95.58/96.55
**TKM-A**	95.43/96.62	95.45/95.41	95.58/96.55	100/100

* ThruPLex/Ultra II

**Fig 4:**
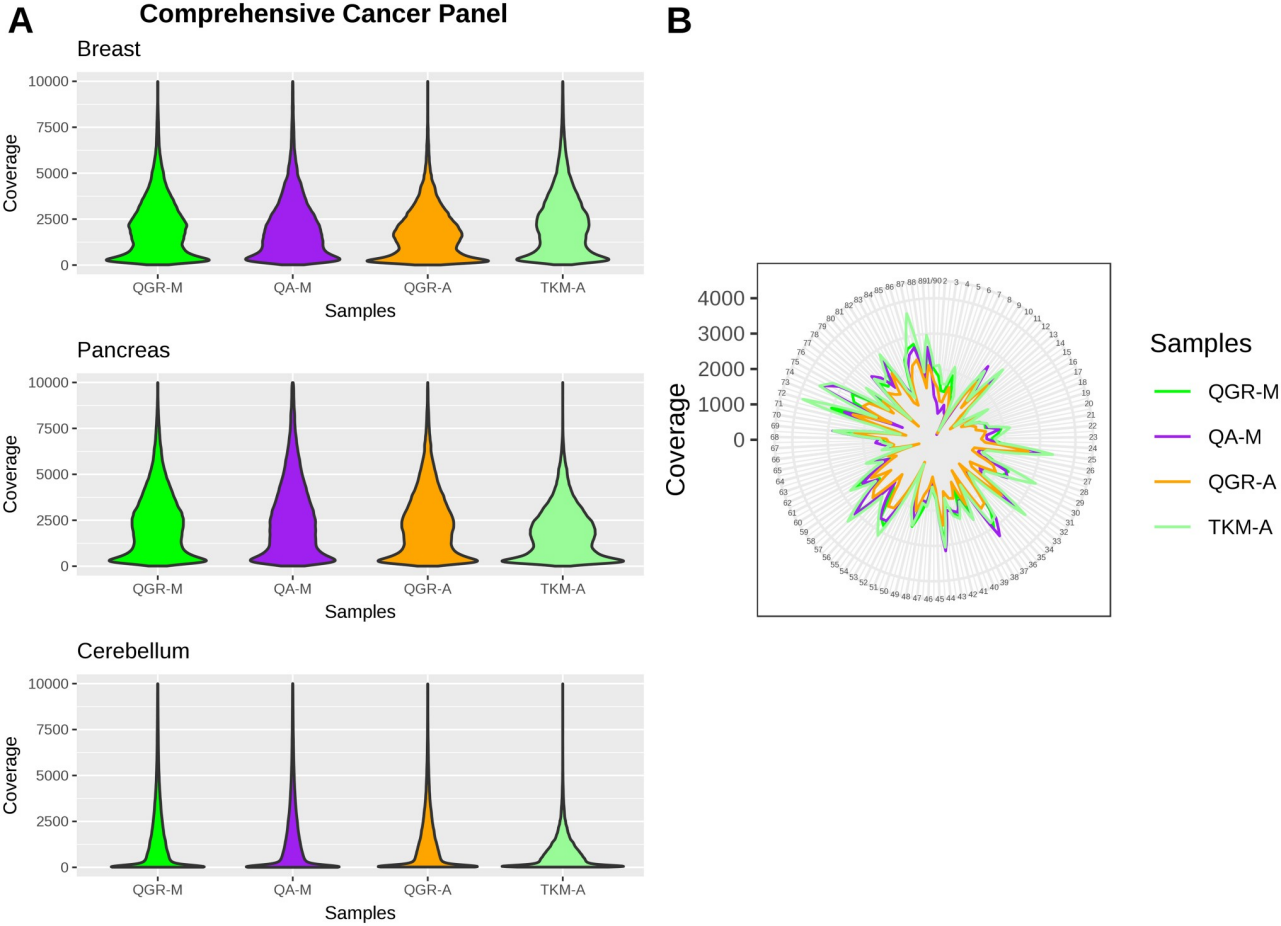
Raw coverage distribution for the four DNA extraction methods. A) Across all targeted bases as seen with the Comprehensive Cancer Panel and B) Across a random subset of the targeted regions in the Comprehensive Cancer Panel. The number 1–98 represent a random set of 98 targeted regions. The CIRCOS plot represents the regions targeted; the coverage lines within are noted on the left (0X the inner most to 4000X the outer most concentric lines).

For targeted sequencing, we evaluated performance in QIAGEN QIAseq Human Comprehensive Cancer (DHS-3501Z) and Human Breast Cancer (DHS-001Z) Panels. Coverage and insert size metrics for each sample are shown in S4 Table. For the Comprehensive Cancer Panel, the highest average molecular tag (MT) coverage of 820.91X was obtained with the TKM-A, while the QGR-M, QA-M and QGR-A methods produced average MT coverages of 650.96X, 599.68X and 733.77X respectively. The distribution of coverage for the comprehensive cancer panel for three representative samples is shown in Fig 4. Each of the four methods yielded some proportion of target bases (even at a raw coverage value) as high as 5000X but the proportion of targeted bases covered above 5000X drops off significantly. For the highly degraded cerebellum sample, the raw coverage drops off beyond 2500X. Fig 4B represents the raw coverage of a subset of targeted regions across the four methods.

Similar coverage results were observed with the Breast Cancer Panel, with the TKM-A method producing the highest average molecular tag coverage of 1609.23X, and the QGR-M, QA-M and QGR-A methods resulting in 749.12X, 1089.31X and 731.02X respectively (data not shown) Table 3 shows the number of variants called with the Comprehensive Cancer Panel and the genotype concordance between the variant calls for the four methods. For calculating genotype concordance between any pair of methods, only the commonly called variants by the pair were considered. The average genotype concordance between the variant calls was found to be high at 99.59% with a standard deviation of s = 0.36 between the four extraction methods.

**Table 3:** 

**Tissue/Method**	**QA-M**	**QGR-M**	**QGR-A**	**TKM-A**
**Number of variants called**				
**Breast**	708	428	507	878
**Pancreas**	1170	493	612	1036
**Cerebellum**	781	427	506	878
**Percent genotype concordance**				
	**QA-M**	**QGR-M**	**QGR-A**	**TKM-A**
**QA-M**	100	99.74	99.75	99.52
**QGR-M**	99.74	100	100	99.03
**QGR-A**	99.75	100	100	99.52
**TKM-A**	99.52	99.03	99.52	100

### Variant analysis and FFPE artefacts

The sequencing data showed high concordance of genotypes between all NGS applications, indicating that any of these four methods could offer optimal yield and quality. Where the sequencing results differ is in variant calling with the Q-GR showing fewer variants called due to the enzymatic repair step which removes artificially induced uracil in the DNA. These artefacts are known to occur in FFPE-derived DNA [1, 25–27], due in part to augmented deamination of cytosine or adenosine [1, 25–28] and show up in sequencing as C>T/G>A or A>G/T>C variants. More recently, Einaga et al[29] and Prentice et al[30] also noted possible artefactual mutations in FFPE samples, highlighting the role of project design and bioinformatics analyses. We observed that the QA-M method called the highest percentage of C>T/G>A mutations at 38.06 percent, while the QGR methods which include the enzymatic repair step called 36.64 percent of variants as C>T/G>A (Table 4).

**Table 4:** WES summary metrics.

	QGR-M	QGR-A	QA-M	TKM-A
**DNA median fragment size**	2644	2146	2270.5	2838
Median % of positions >20X*	47.65/61.67	40.96/61.00	44.04/63.70	43.67/52.75
Median coverage*	19.5/24.5	17/23.75	18.5/25.75	18/20.75
% duplicated reads*	18/9.6	19.6/14	22.2/14.4	19.2/9.6
**C.T>G.A rate for SNV (% C>T/G>A mutations)**	36.64	37.5	38.06	36.59

*with/without cerebellum

**Fig 5:**
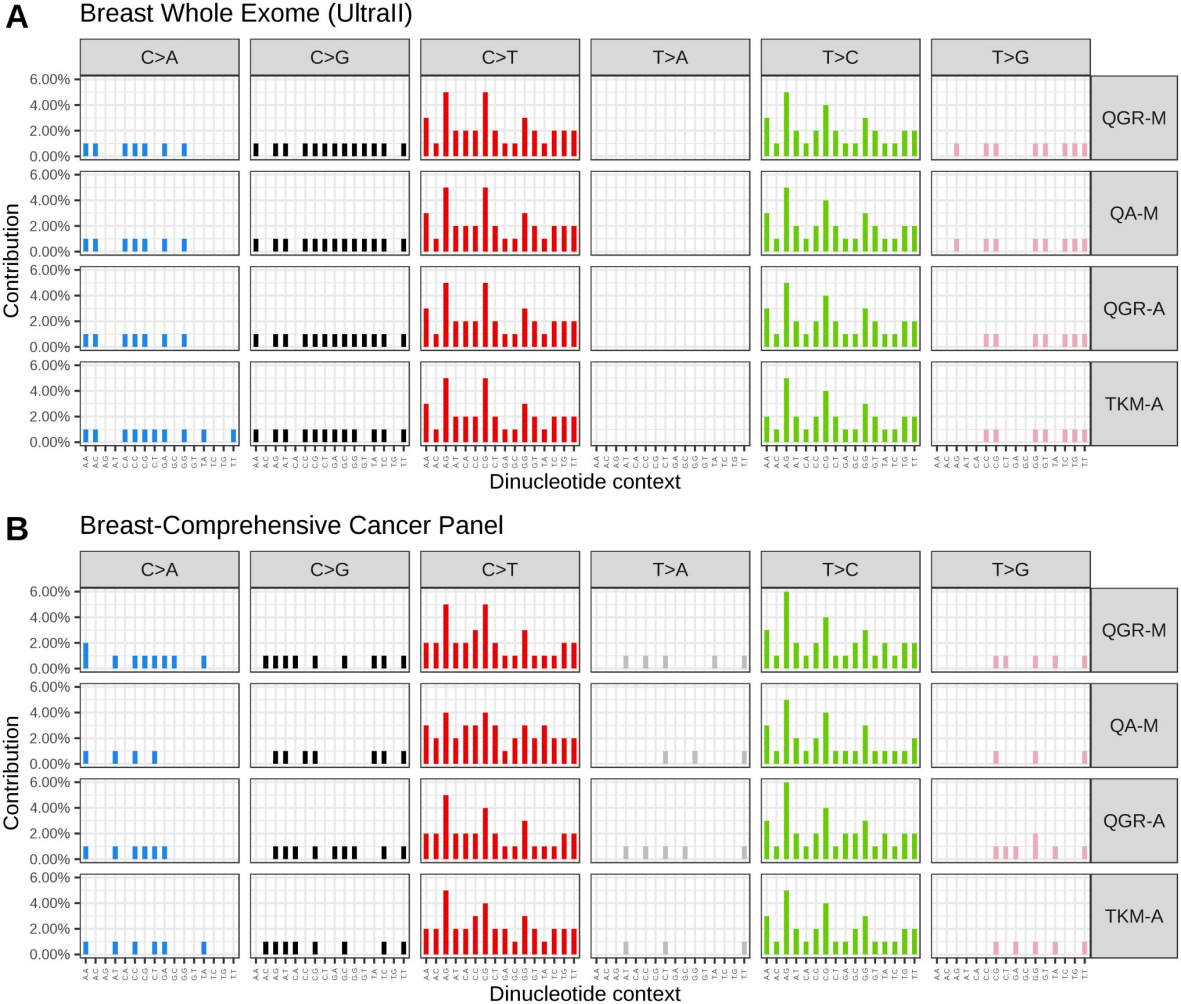
Signature of mutations found in the FFPE breast samples. A) Standard whole exome sequencing (UltraII) and B) Targeted Comprehensive Cancer Panel. A large proportion of the mutations are seen in the C>T context resulting from cytosine de-aminations in both datasets. The QGR method involving the repair step reduces but does not eliminate these C>T mutations.

When looking at the sequence context of the C>T transitions[18], we found 43% to occur in CpG dyads. Additionally, we observed that ~32% of variants were also called as T>C/G>A, which have also been reported as artefacts resulting from FFPE DNA[26]. Fig 5A further shows the distribution of variant signatures seen across the Breast FFPE sample in whole exome sequencing (data only shown for the UltraII method); Fig 5B shows the same with the Comprehensive Cancer Panel; S3 Fig contains FFPE signatures for all samples and methods.

## Discussion

DNA extraction methods for FFPE tissues vary in quality and quantity of resultant DNA, all of which may impact performance in downstream assays. In this report, we evaluated nine methods, including both manual and automated protocols, as the latter are preferred to minimize potential errors in sample handling. QC evaluations were performed to determine the degree of DNA damage and which methods application might procure the best quality data; DNA from four extraction methods (manual and automated) were assayed for low input WES and amplicon based targeted sequencing based on yield, percent dsDNA, DQN and fragment length. Three of these methods, QIAGEN GeneRead (GR-A or GR-M), Promega Maxwell (PM-A) and QIAGEN QIAamp (QA-M or QA-A), were evaluated in Bonnet et al[5]; QA and GR were less fragmented than PM-A, having longer median fragment lengths, in agreement with findings in this report.

For WES, the NEBNext Ultra II low input library preparation provided lower duplicated reads, better coverage, and higher reads in the capture regions than did those prepared using ThruPlex for the four extraction methods. The very fragmented DNA from cerebellum had lower reads in the capture regions and higher duplication rates for WES but was effectively profiled using an amplicon-based targeted approach Unlike Bonnet et al[5] who observed an approximate average median coverage difference of 13.5X between extraction methods for FFPE samples, we observed that both GR-A/M and QA-M methods produced similar metrics between the four extraction methods with respect to coverage (Δ_coverage_ = 2.55X), percentage of bases with ≥ 20x coverage (Δ_pctbases ≥ 20X_ = 5.05 percent), percent bases mapping in target (Δ_target_ = 4.25 percent) and outside target regions (Δ_off target_ = 3.12 percent). However, it should be noted that we used a low DNA input (50ng) rather than 200ng used by Bonnet et al.

The low input library preparation methods were very similar, the only difference being the use of SPRIselect beads (Beckman Coulter, Indianapolis, IN, USA) which help to contain library size, suggesting that small changes can impact downstream performance. Small input NGS applications are constantly evolving and include improvements to enzymatics, molecular barcoding and bead based technologies which should further broaden the number of applications available for use with FFPE and other small input sample sources (https://www.qiagen.com/us/resources/resourcedetail?id=b3363886-aaed-4e0d-8d4b-3291b28593c5&lang=en; https://support.illumina.com/content/dam/illumina-marketing/documents/products/datasheets/nextera-dna-flex-data-sheet-770-2017-011.pdf). There are some challenges using FFPE DNA in NGS, particularly when the quantity of DNA is limited. There were no substantial differences in sequencing results for the four selected protocols, neither was there any difference in the spectrum of variation found. The FFPE signature, of C>T transitions was similar in all tested methods. As in Spencer et al [18], the finding that C>T transitions often occurred in CpG dyads supports the observation that deamination of cytosine FFPE is a major source of artefactual variations in FFPE DNA[1]. This occurs in living cells and uracil-DNA glycosylase (UDG) removes the altered base; the abasic site is then restored to cytosine by base excision repair[1]. The GeneRead protocol includes a repair step (UDG) which ameliorates, but does not totally eliminate, these artefacts. Bioinformatics approaches to dealing with these artefacts will enhance the use of FFPE derived DNA in epidemiologic studies.

The strengths of this report are the evaluation of nine DNA extraction methods, including manual and automated, and evaluation of low input library preparation protocols. Sequential sections from a single block for each sample were used to best assess each extraction approach. As improved techniques in FFPE extractions become available comparisons to standard laboratory protocols must be made to determine whether these changes have an impact on downstream analysis. This is also true for small input NGS applications where improvements to enzymatics, enrichment methods, and bead based technologies have made it possible to reduce workflow, increase sensitivity, and lower input amounts to as low as 1.0ng. With continued improvements in both methods it has become possible to include poor quality, low yield FFPE samples, further broadening the number of low input samples for study.

## Supporting information

S1 Fig Average 260/280 ratios of the 12 DNAs for each extraction method. (DOCX)

S2 Fig Heat map showing the percentage of target bases covered by pancreas tumor, cerebellum and breast cancer samples, DNA extraction and library preparation methods. (DOCX)

S3 Fig FFPE Signature Distribution for A)Breast WES (Thruplex) B)Cerebellum WES (Thruplex) C) Cerebellum WES (UltraII) D) Cerebellum Targeted (Comprehensive Cancer Panel) E) Pancreas WES (Thruplex) F) Pancreas WES (UltraII) G) Pancreas Targeted (Comprehensive Cancer Panel). (DOCX)

S1 Table Percentage of fragments within each size range for all DNA extraction methods for the 12 samples. (DOCX)

S2 Table Multiplex PCR data expressed as percentage compared to a CEPH control. Highlighted columns represent those methods selected for evaluation in NGS.
(DOCX)

S3 Table Whole exome sequencing metrics for the two library preparation methods and four selected DNA extraction methods. (DOCX)

S4 Table Metrics for the two targeted NGS panels. (DOCX)
