# Supplementary material for: Use of FFPE-derived DNA in next generation sequencing: DNA extraction methods
Source: PLoS One. 2019 Apr 11;14(4):e0211400. doi: 10.1371/journal.pone.0211400 (PMC6459541; doi:10.1371/journal.pone.0211400)
Supplement: S1 Fig — (DOCX) [file pone.0211400.s001.docx]

**S1 Figure**  Distribution of 260/280 ratios of the 12 DNAs for each DNA extraction method
