# Supplementary material for: Use of FFPE-derived DNA in next generation sequencing: DNA extraction methods
Source: PLoS One. 2019 Apr 11;14(4):e0211400. doi: 10.1371/journal.pone.0211400 (PMC6459541; doi:10.1371/journal.pone.0211400)
Supplement: S2 Fig — (DOCX) [file pone.0211400.s002.docx]

**S2 Figure** Heat map showing the percentage of target bases covered by pancreas tumor, cerebellum and breast cancer samples with **A)** Thruplex **B)** Ultra II

**A**

**
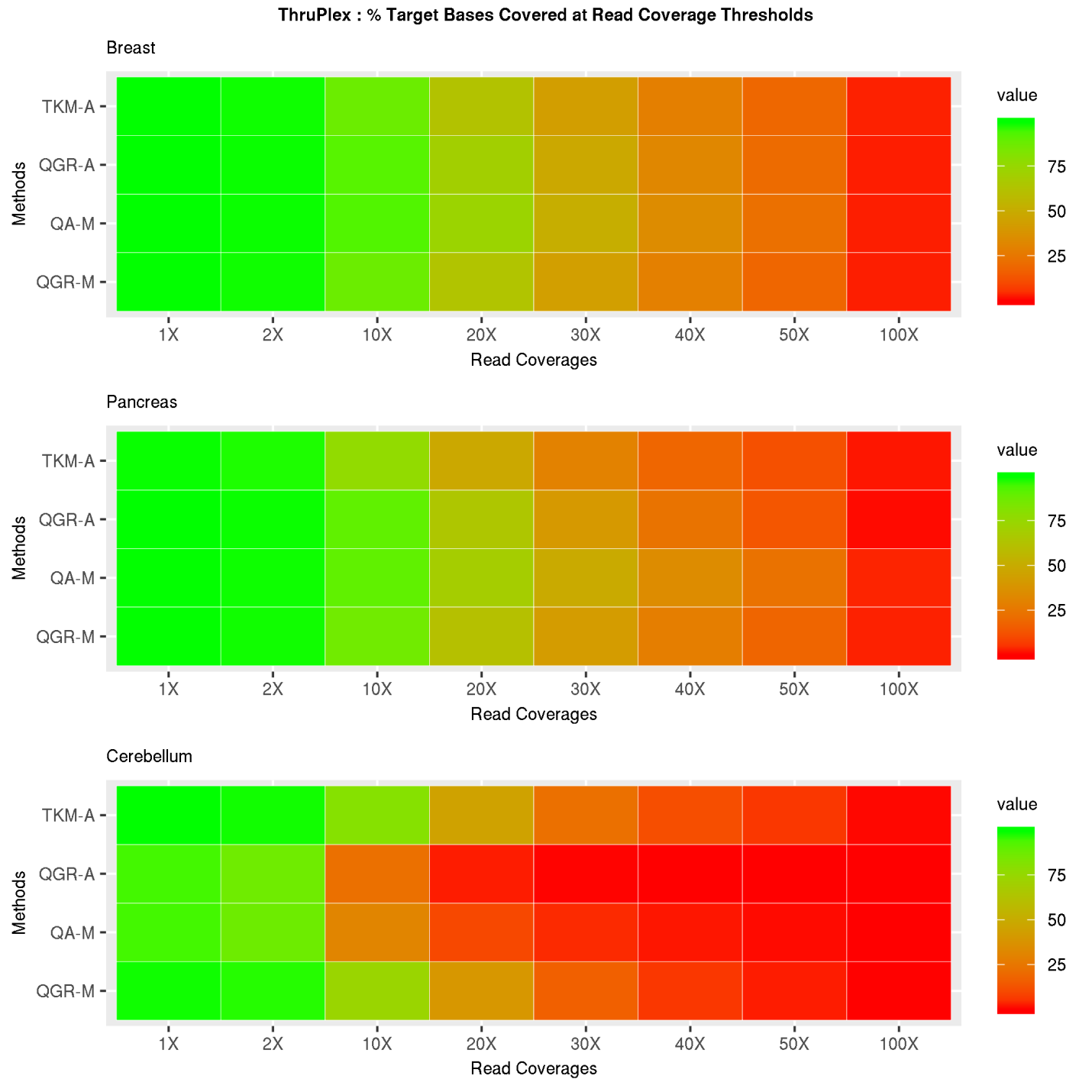
**

**B**

**
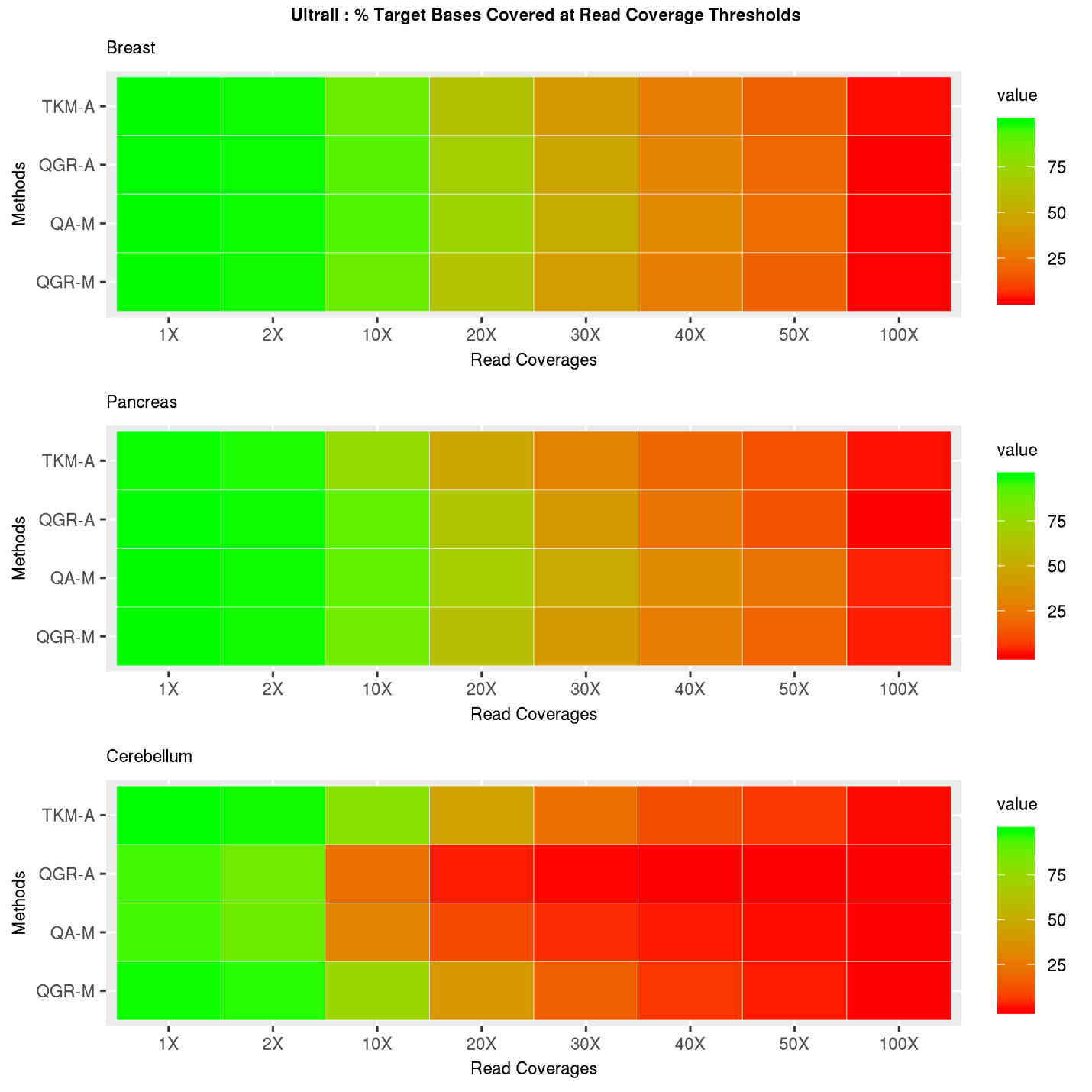
**
