# Supplementary figures and images for: Use of FFPE-derived DNA in next generation sequencing: DNA extraction methods
Source: PLoS One. 2019 Apr 11;14(4):e0211400. doi: 10.1371/journal.pone.0211400 (PMC6459541; doi:10.1371/journal.pone.0211400)

**S3. FFPE Signatures for Samples and Methods**

| **A**  **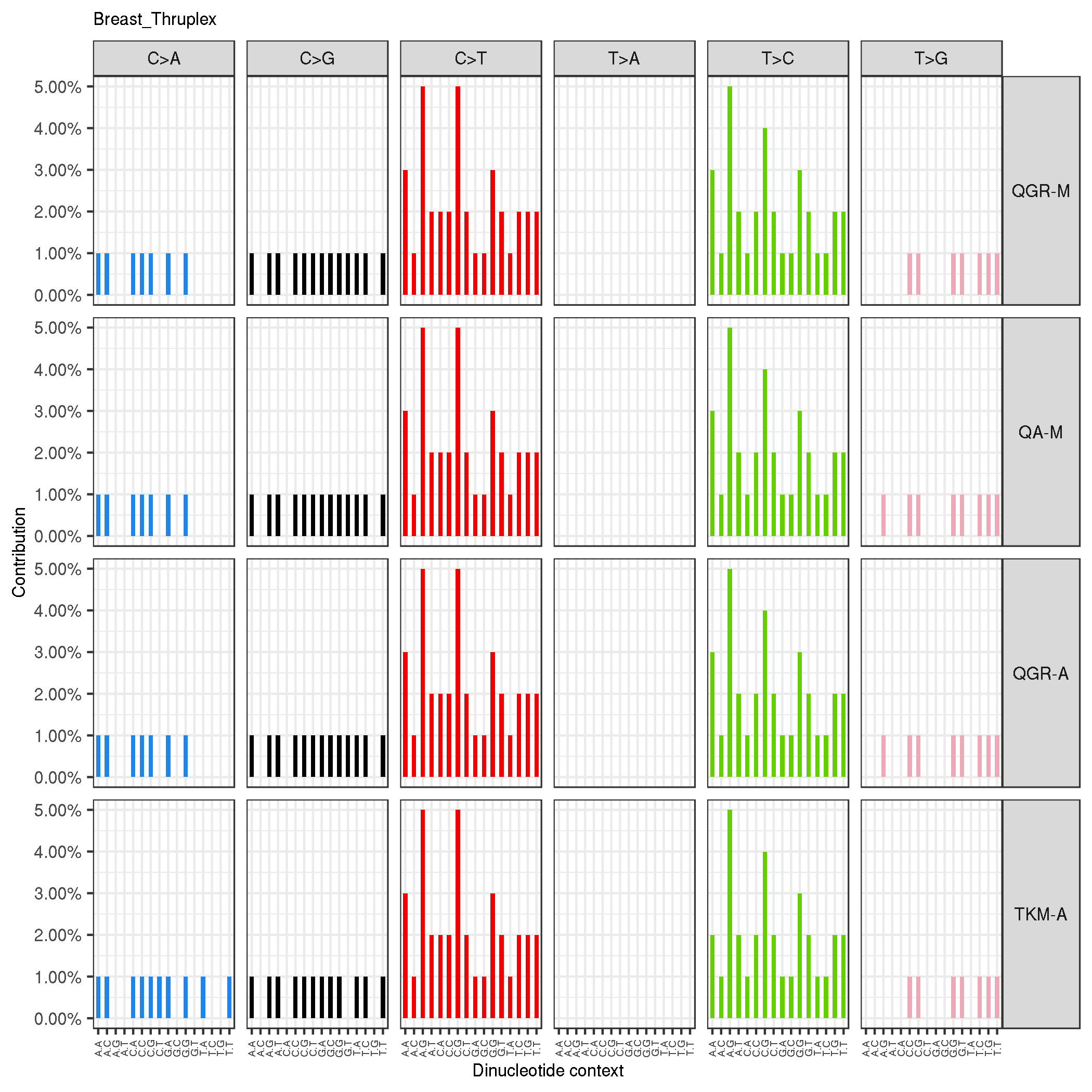** | **B**  **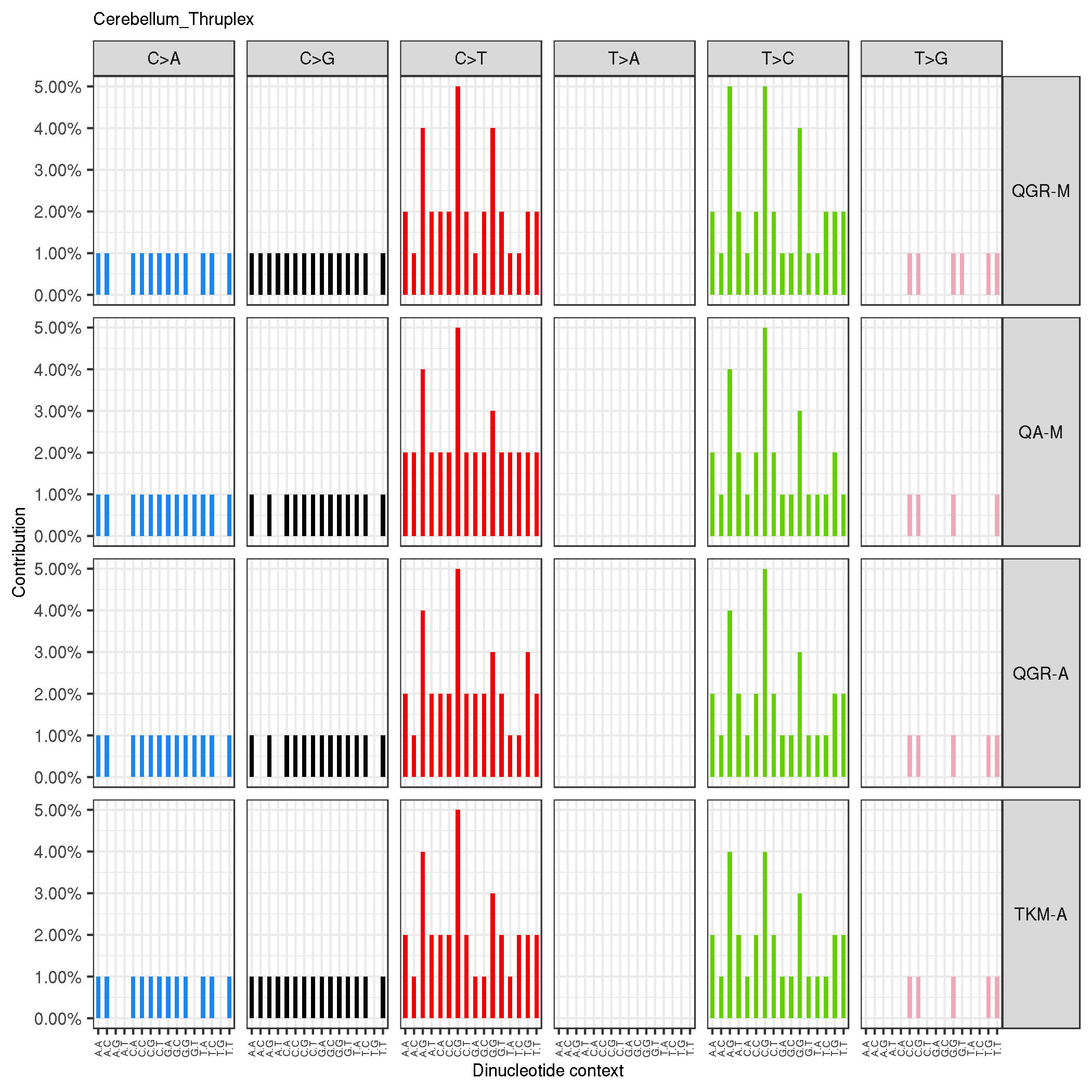** |
| --- | --- |
| **C**  **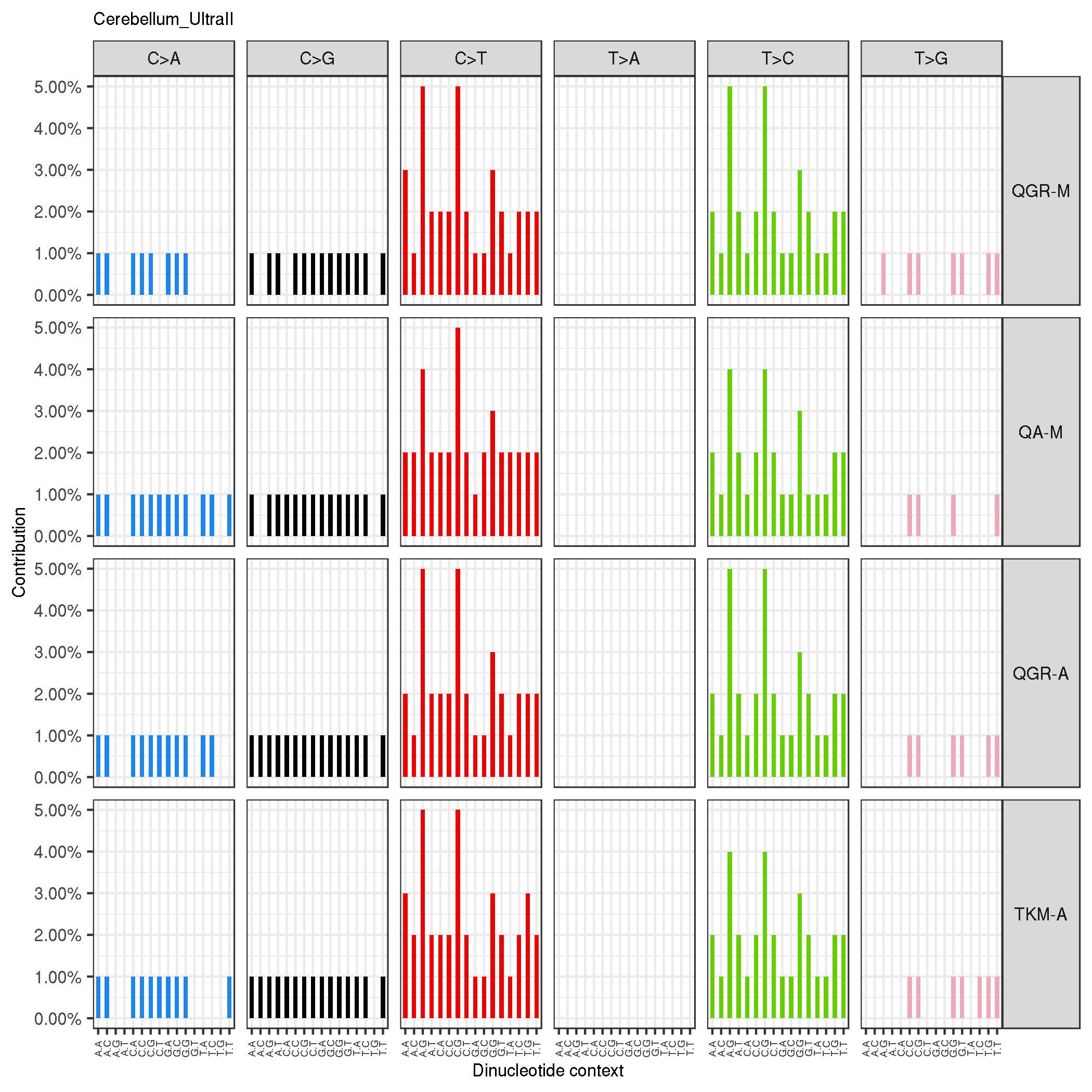** | **D**  **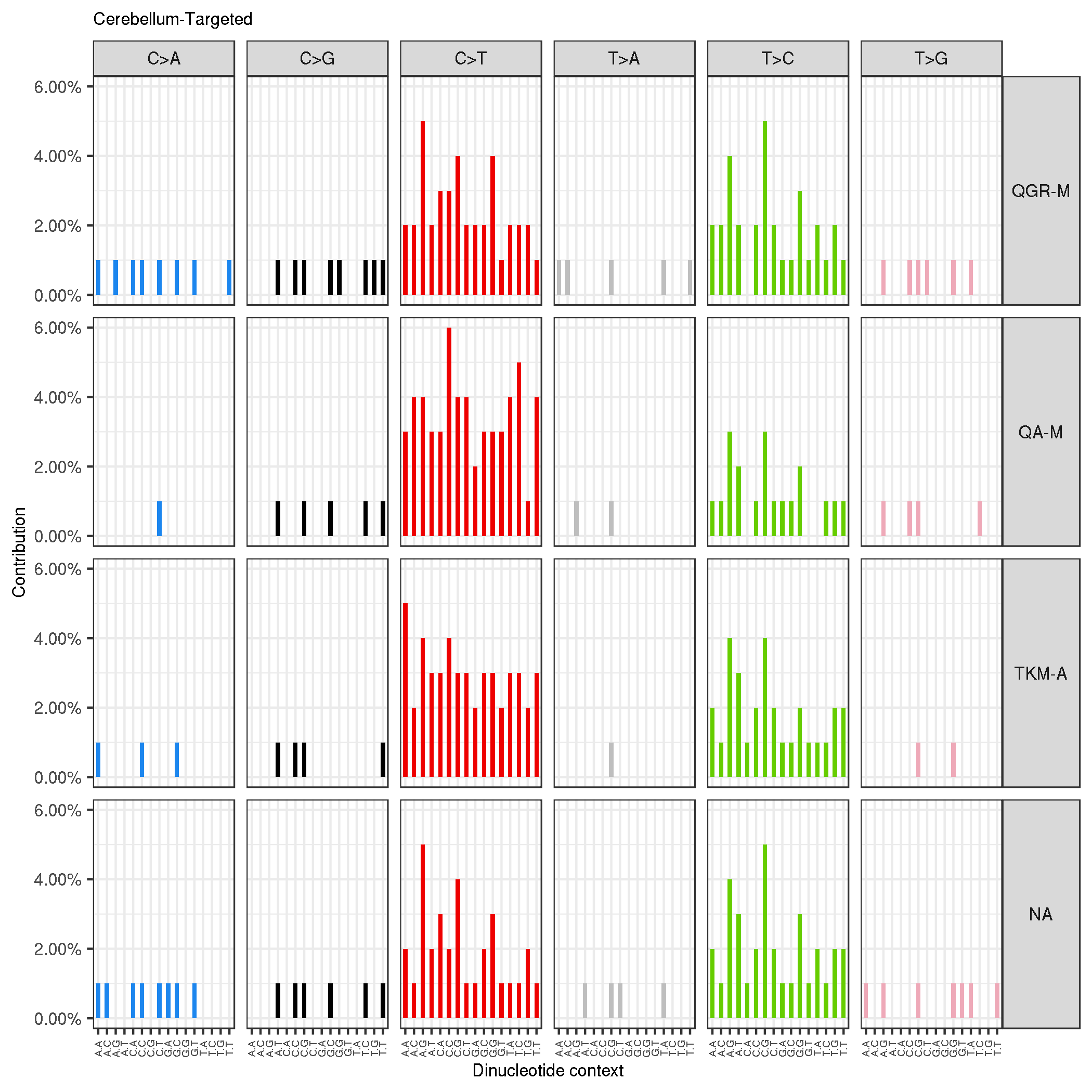** |

| **E**  **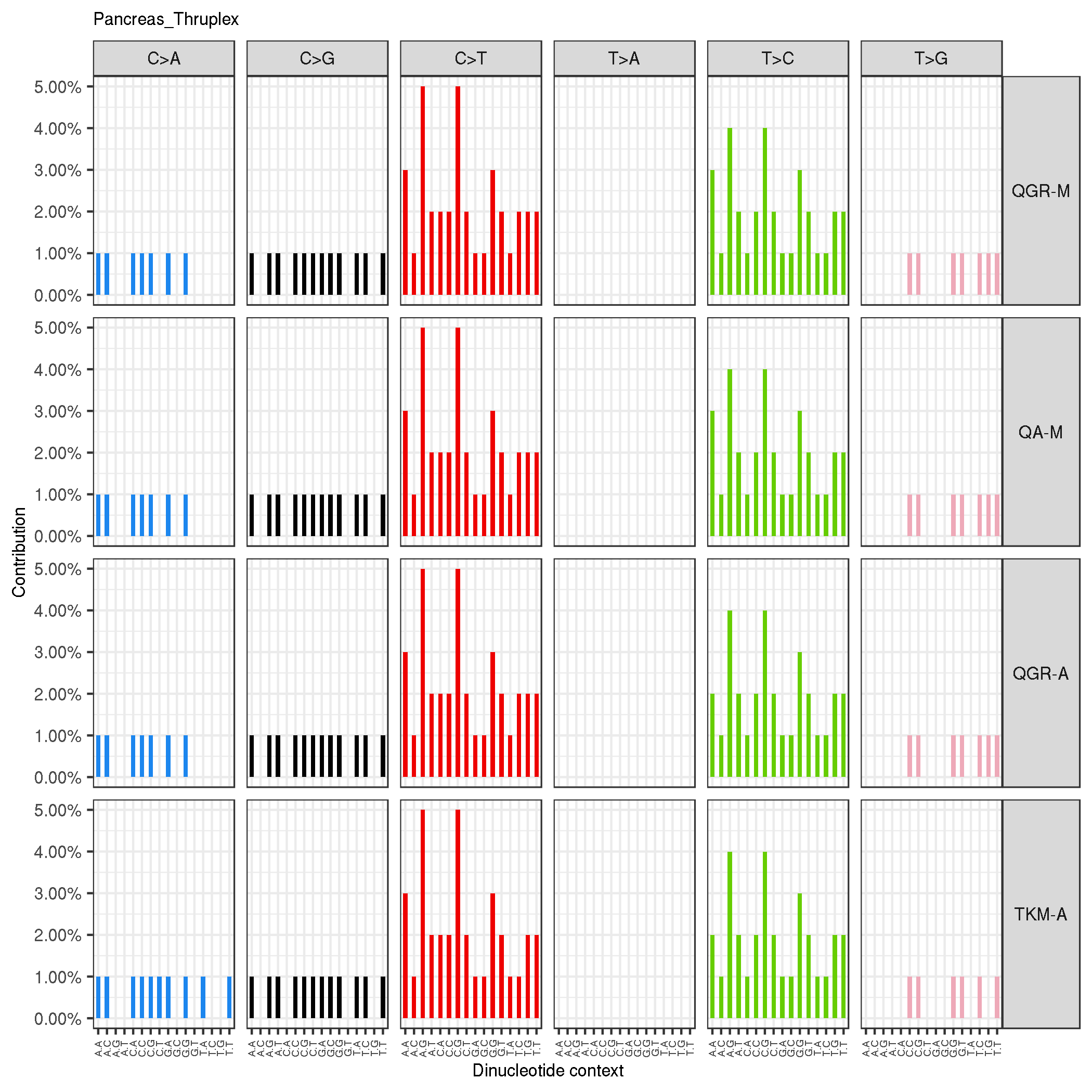** | **F**  **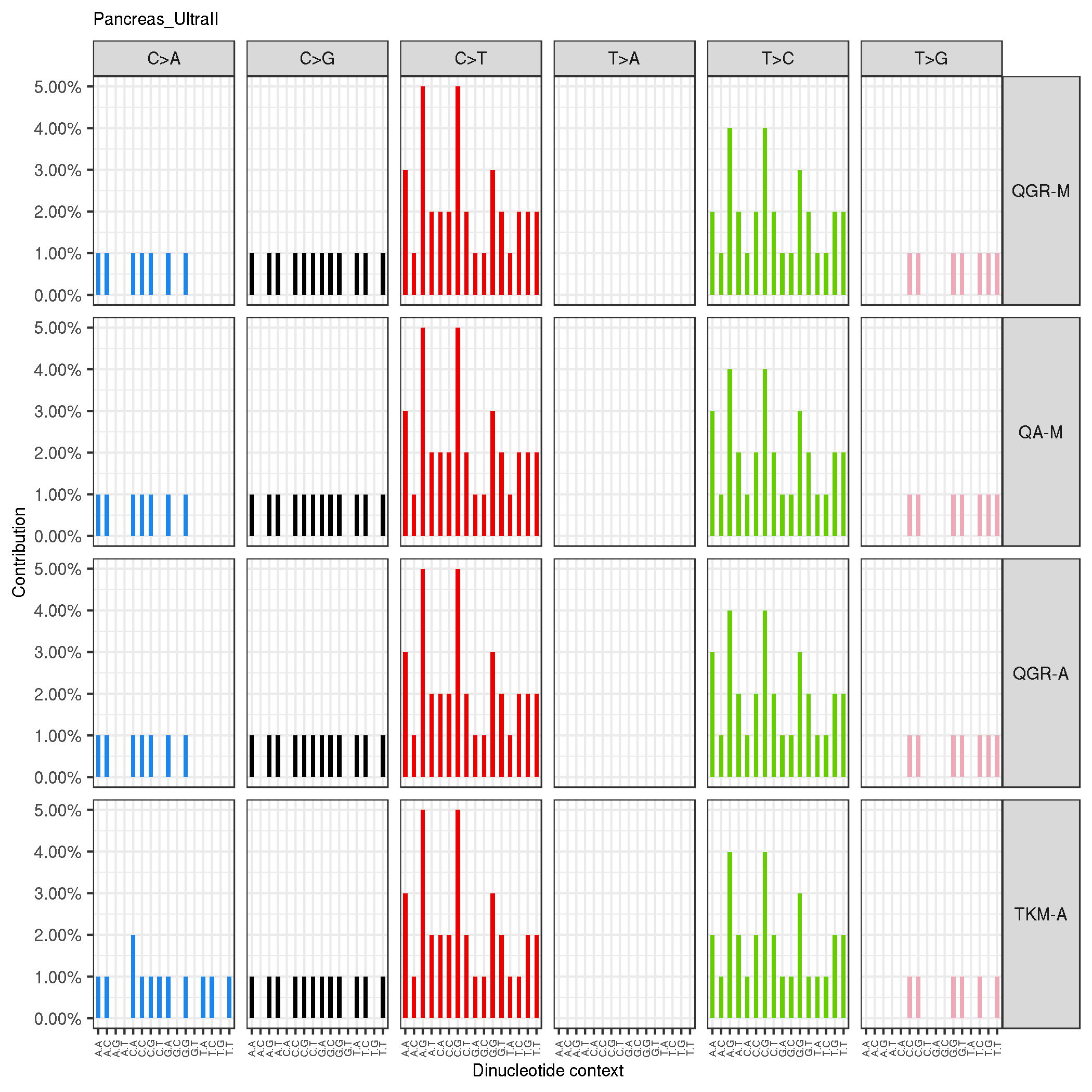** |
| --- | --- |
| **G**  **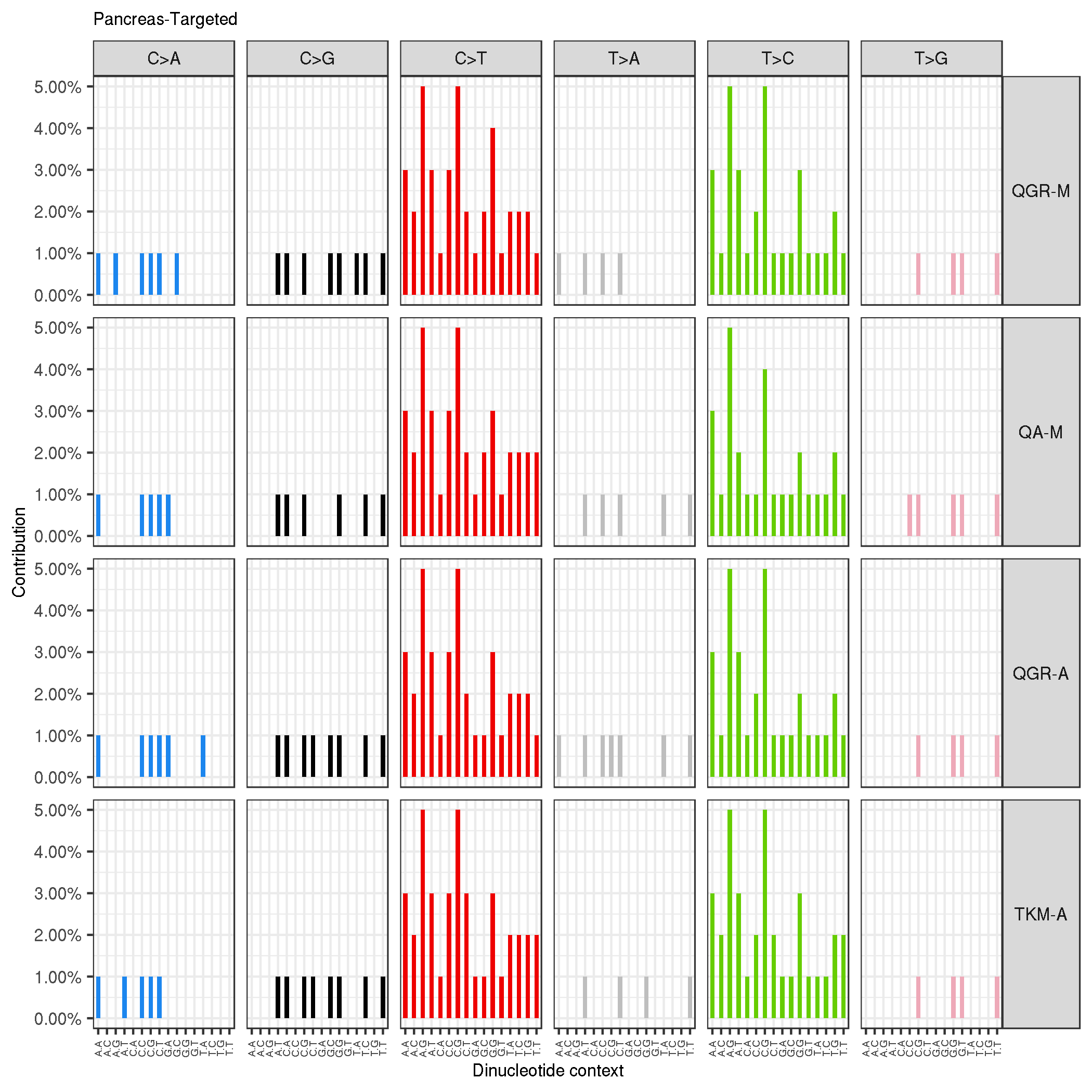** |

Supplement: S3 Fig — (DOCX) [file pone.0211400.s003.docx]
