# Supplementary material for: Use of FFPE-derived DNA in next generation sequencing: DNA extraction methods
Source: PLoS One. 2019 Apr 11;14(4):e0211400. doi: 10.1371/journal.pone.0211400 (PMC6459541; doi:10.1371/journal.pone.0211400)
Supplement: S1 Table — (DOCX) [file pone.0211400.s004.docx]

**S1 Table**  Percentage of fragments within each size range for all DNA extraction methods for the 12 samples.

|  | **KE-M** | **PR-M** | **QGR-M** | **QA-M** | **QS-A** | **QGR-A** | **PM-A** | **PEC-A** | **TKM-A** |
| --- | --- | --- | --- | --- | --- | --- | --- | --- | --- |
| **Size Range (bp)** | **% Total** | **% Total** | **% Total** | **% Total** | **% Total** | **% Total** | **% Total** | **% Total** | **% Total** |
| **Breast N 1- 200 bp** | 71.0 | 69.9 | 21.2 | 13.7 | 66.7 | 15.7 | 38.1 | 61.1 | 7.5 |
| **Breast N 201- 1000 bp** | 13.1 | 13.7 | 37.3 | 40.6 | 31.3 | 48.2 | 48.6 | 31.2 | 29.5 |
| **Breast N 1001- 20000 bp** | 15.9 | 16.4 | 41.5 | 45.8 | 1.9 | 36.0 | 13.3 | 7.7 | 63.0 |
| **Breast T 1 - 200 bp** | 70.1 | 44.9 | 8.5 | 20.5 | 81.2 | 11.3 | 18.2 | 63.3 | 5.1 |
| **Breast T 201 -1000 bp** | 11.5 | 39.5 | 37.3 | 29.9 | 18.8 | 46.8 | 46.7 | 21.8 | 40.8 |
| **Breast T 1001- 20000 bp** | 18.4 | 15.6 | 54.2 | 49.6 | 0.0 | 41.9 | 35.1 | 14.8 | 54.1 |
| **Colon N 1 - 200 bp** | 76.1 | 35.3 | 6.9 | 25.3 | 10.1 | 9.7 | 22.8 | 72.0 | 6.6 |
| **Colon N 201 - 1000 bp** | 8.7 | 43.8 | 43.3 | 27.0 | 27.7 | 48.6 | 51.0 | 18.2 | 40.2 |
| **Colon N 1001 - 20000 bp** | 15.2 | 20.9 | 49.8 | 47.7 | 62.2 | 41.7 | 26.2 | 9.8 | 53.1 |
| **Colon T 1 - 200 bp** | 43.0 | 22.7 | 7.6 | 19.2 | 15.2 | 12.5 | 24.1 | 25.9 | 6.3 |
| **Colon T 201 - 1000 bp** | 24.1 | 48.0 | 53.8 | 48.4 | 30.0 | 58.2 | 52.3 | 45.6 | 51.3 |
| **Colon T 1001 - 20000 bp** | 32.9 | 29.3 | 38.7 | 32.3 | 54.9 | 29.3 | 23.6 | 28.5 | 42.4 |
| **Lung N 1- 200 bp** | 80.0 | 33.7 | 16.5 | 19.7 | 17.5 | 10.8 | 22.7 | 70.0 | 6.2 |
| **Lung N 201- 1000 bp** | 10.9 | 48.0 | 33.1 | 33.2 | 42.6 | 40.9 | 52.9 | 22.1 | 41.3 |
| **Lung N 1001 - 20000 bp** | 9.1 | 18.3 | 50.4 | 47.1 | 39.9 | 48.3 | 24.4 | 7.8 | 52.5 |
| **Lung T 1 - 200 bp** | 64.1 | 31.0 | 11.8 | 31.8 | 35.4 | 15.0 | 31.0 | 43.8 | 13.1 |
| **Lung T 201-1000 bp** | 21.9 | 48.2 | 55.8 | 43.3 | 33.9 | 59.2 | 53.0 | 43.6 | 46.9 |
| **Lung T 1001- 20000 bp** | 14.1 | 20.8 | 32.4 | 24.9 | 30.7 | 25.8 | 16.0 | 12.6 | 40.0 |
| **Pancreas N 1 - 200 bp** | 74.3 | 48.2 | 7.1 | 30.5 | 20.1 | 12.6 | 22.9 | 38.6 | 15.4 |
| **Pancreas N 201- 1000 bp** | 11.7 | 30.5 | 50.4 | 46.9 | 44.9 | 55.2 | 52.6 | 50.0 | 55.7 |
| **Pancreas N 1001- 20000 bp** | 14.1 | 21.3 | 42.6 | 22.6 | 35.1 | 32.1 | 24.4 | 11.4 | 28.9 |
| **Pancreas T 1 -200 bp** | 46.9 | 36.8 | 4.6 | 23.3 | 16.7 | 11.0 | 20.1 | 41.1 | 5.3 |
| **Pancreas T 201 - 1000 bp** | 18.9 | 43.4 | 46.2 | 34.8 | 38.7 | 48.7 | 54.8 | 31.4 | 46.8 |
| **Pancreas T 1001- 20000 bp** | 34.2 | 19.8 | 49.2 | 41.9 | 44.6 | 40.2 | 25.1 | 27.5 | 47.9 |
| **Brain Stem 1 - 200 bp** | 47.5 | 56.6 | 52.5 | 46.1 | 49.4 | 54.7 | 61.0 | 35.0 | 38.5 |
| **Brain Stem 201- 1000 bp** | 49.1 | 42.7 | 46.5 | 52.2 | 49.6 | 44.6 | 38.5 | 63.4 | 59.4 |
| **Brain Stem 1001 -20000 bp** | 3.4 | 0.7 | 1.0 | 1.7 | 1.1 | 0.7 | 0.5 | 1.6 | 2.1 |
| **Cerebellum 1 - 200 bp** | 46.6 | 51.4 | 46.4 | 39.6 | 34.7 | 48.7 | 56.0 | 21.6 | 24.5 |
| **Cerebellum 201- 1000 bp** | 51.3 | 47.8 | 52.9 | 59.5 | 63.4 | 50.8 | 42.2 | 72.4 | 73.4 |
| **Cerebellum 1001 - 20000 bp** | 2.1 | 0.8 | 0.7 | 0.9 | 1.9 | 0.5 | 1.9 | 6.0 | 2.1 |
| **Tonsil 1 1 - 200 bp** | 42.6 | 26.8 | 8.5 | 17.5 | 17.5 | 11.4 | 26.9 | 32.8 | 10.0 |
| **Tonsil 1 201 - 1000 bp** | 38.8 | 55.8 | 59.4 | 55.2 | 50.8 | 62.5 | 60.5 | 53.5 | 56.6 |
| **Tonsil 1 1001- 20000 bp** | 18.6 | 17.3 | 32.0 | 27.3 | 31.7 | 26.1 | 12.6 | 13.7 | 33.4 |
| **Tonsil 2 1- 200 bp** | 46.3 | 22.6 | 9.2 | 21.9 | 16.6 | 14.4 | 31.4 | 26.4 | 11.9 |
| **Tonsil 2 201- 1000 bp** | 38.7 | 61.1 | 62.6 | 56.0 | 57.0 | 63.0 | 57.9 | 55.8 | 58.7 |
| **Tonsil 2 1001 - 20000 bp** | 15.1 | 16.3 | 28.3 | 22.1 | 26.4 | 22.6 | 10.7 | 17.8 | 29.4 |
