# Supplementary material for: Use of FFPE-derived DNA in next generation sequencing: DNA extraction methods
Source: PLoS One. 2019 Apr 11;14(4):e0211400. doi: 10.1371/journal.pone.0211400 (PMC6459541; doi:10.1371/journal.pone.0211400)
Supplement: S2 Table — Highlighted columns represent those methods selected for evaluation in NGS. (DOCX) [file pone.0211400.s005.docx]

**S2 Table**  Multiplex PCR data expressed as percentage compared to a CEPH control. Highlighted columns represent those methods selected for evaluation in NGS.

|  | **Ratio of Extracted vs Control Sample** | | | | | | | | | | |  |  |
| --- | --- | --- | --- | --- | --- | --- | --- | --- | --- | --- | --- | --- | --- |
| **Extracted FFPE Tissue** | | | **Amplicon Size (bp)** | **KE-M** | **PR-M** | **QGR-M** | **QA-M** | **QS-A** | **QGR-A** | **PM-A** | **PEC-A** | **TKM-A** | |
|  | | | **132** | 64% | 113% | 108% | 46% | 0% | 51% | 43% | 1% | 70% | |
| **Breast** | | | **150** | 23% | 21% | 72% | 23% | 0% | 31% | 7% | 0% | 49% | |
| **Normal** | | | **196** | 33% | 70% | 69% | 31% | 1% | 26% | 16% | 0% | 59% | |
|  | | | **235** | 18% | 20% | 44% | 27% | 1% | 14% | 9% | 0% | 50% | |
|  | | | **295** | 8% | 5% | 31% | 21% | 0% | 11% | 4% | 2% | 27% | |
|  | | | **132** | 92% | 170% | 147% | 88% | 81% | 48% | 67% | 22% | 96% | |
| **Breast** | | | **150** | 44% | 60% | 98% | 34% | 41% | 34% | 25% | 19% | 60% | |
| **Tumor** | | | **196** | 53% | 104% | 101% | 47% | 63% | 32% | 36% | 13% | 76% | |
|  | | | **235** | 34% | 48% | 74% | 39% | 56% | 29% | 30% | 12% | 68% | |
|  | | | **295** | 15% | 21% | 46% | 24% | 25% | 17% | 16% | 3% | 37% | |
|  | | | **132** | 64% | 122% | 92% | 89% | 66% | 86% | 140% | 20% | 95% | |
| **Colon** | | | **150** | 24% | 30% | 58% | 35% | 20% | 56% | 34% | 15% | 59% | |
| **Normal** | | | **196** | 35% | 72% | 59% | 50% | 47% | 48% | 60% | 12% | 71% | |
|  | | | **235** | 21% | 27% | 36% | 40% | 35% | 40% | 35% | 10% | 58% | |
|  | | | **295** | 12% | 12% | 27% | 27% | 19% | 29% | 23% | 4% | 38% | |
|  | | | **132** | 113% | 116% | 175% | 87% | 46% | 94% | 123% | 24% | 79% | |
| **Colon** | | | **150** | 74% | 61% | 143% | 63% | 43% | 73% | 67% | 25% | 73% | |
| **Tumor** | | | **196** | 71% | 77% | 115% | 58% | 38% | 53% | 77% | 16% | 73% | |
|  | | | **235** | 55% | 48% | 86% | 49% | 44% | 45% | 59% | 15% | 72% | |
|  | | | **295** | 34% | 31% | 71% | 42% | 24% | 36% | 40% | 9% | 47% | |
|  | | | **132** | 52% | 140% | 127% | 122% | 42% | 98% | 166% | 11% | 99% | |
| **Lung** | | | **150** | 21% | 34% | 66% | 31% | 12% | 71% | 35% | 1% | 61% | |
| **Normal** | | | **196** | 28% | 89% | 83% | 52% | 33% | 61% | 75% | 0% | 72% | |
|  | | | **235** | 20% | 40% | 59% | 41% | 31% | 56% | 54% | 0% | 66% | |
|  | | | **295** | 11% | 13% | 42% | 23% | 11% | 40% | 20% | 0% | 43% | |
|  | | | **132** | 94% | 118% | 110% | 75% | 34% | 90% | 107% | 20% | 64% | |
| **Lung** | | | **150** | 66% | 62% | 94% | 59% | 34% | 81% | 64% | 29% | 64% | |
| **Tumor** | | | **196** | 59% | 86% | 82% | 54% | 38% | 55% | 66% | 15% | 72% | |
|  | | | **235** | 45% | 42% | 48% | 44% | 39% | 37% | 47% | 13% | 62% | |
|  | | | **295** | 21% | 19% | 35% | 29% | 16% | 25% | 26% | 6% | 42% | |
|  | | | **132** | 79% | 114% | 115% | 94% | 36% | 97% | 126% | 22% | 79% | |
| **Pancreas** | | | **150** | 50% | 51% | 87% | 59% | 29% | 79% | 64% | 24% | 65% | |
| **Normal** | | | **196** | 54% | 79% | 82% | 55% | 31% | 62% | 78% | 16% | 62% | |
|  | | | **235** | 38% | 42% | 58% | 47% | 37% | 52% | 58% | 14% | 57% | |
|  | | | **295** | 22% | 25% | 43% | 31% | 16% | 36% | 37% | 7% | 35% | |
|  | | | **132** | 79% | 32% | 118% | 70% | 48% | 99% | 123% | 27% | 74% | |
| **Pancreas** | | | **150** | 40% | 8% | 84% | 36% | 39% | 77% | 50% | 25% | 56% | |
| **Tumor** | | | **196** | 44% | 22% | 81% | 42% | 41% | 62% | 76% | 19% | 56% | |
|  | | | **235** | 28% | 8% | 58% | 31% | 48% | 50% | 52% | 15% | 48% | |
|  | | | **295** | 24% | 3% | 41% | 25% | 25% | 41% | 35% | 8% | 33% | |
|  | | | **132** | 4% | 37% | 42% | 2% | 0% | 4% | 8% | 0% | 19% | |
| **Brain Stem** | | | **150** | 0% | 15% | 22% | 1% | 0% | 2% | 9% | 0% | 15% | |
|  | | | **196** | 2% | 9% | 11% | 0% | 0% | 0% | 3% | 0% | 15% | |
|  | | | **235** | 1% | 3% | 4% | 0% | 0% | 0% | 1% | 0% | 9% | |
|  | | | **295** | 0% | 1% | 1% | 0% | 0% | 0% | 0% | 0% | 5% | |
|  | | | **132** | 7% | 28% | 21% | 3% | 0% | 4% | 15% | 1% | 18% | |
| **Cerebellum** | | | **150** | 2% | 23% | 19% | 4% | 1% | 2% | 18% | 2% | 19% | |
|  | | | **196** | 1% | 10% | 5% | 0% | 0% | 0% | 4% | 0% | 11% | |
|  | | | **235** | 1% | 1% | 2% | 0% | 0% | 0% | 2% | 0% | 6% | |
|  | | | **295** | 1% | 2% | 1% | 0% | 0% | 0% | 1% | 0% | 2% | |
|  | | | **132** | 28% | 109% | 97% | 35% | 10% | 36% | 97% | 17% | 43% | |
| **Tonsil 1** | | | **150** | 8% | 49% | 63% | 25% | 9% | 25% | 39% | 15% | 37% | |
|  | | | **196** | 10% | 66% | 56% | 20% | 7% | 17% | 48% | 9% | 32% | |
|  | | | **235** | 4% | 27% | 32% | 16% | 8% | 13% | 28% | 5% | 24% | |
|  | | | **295** | 3% | 11% | 16% | 7% | 2% | 4% | 10% | 2% | 15% | |
|  | | | **132** | 64% | 110% | 90% | 42% | 17% | 54% | 78% | 12% | 42% | |
| **Tonsil 2** | | | **150** | 26% | 48% | 58% | 29% | 12% | 35% | 39% | 9% | 34% | |
|  | | | **196** | 29% | 61% | 54% | 25% | 10% | 25% | 34% | 6% | 33% | |
|  | | | **235** | 15% | 24% | 27% | 19% | 11% | 18% | 22% | 4% | 25% | |
|  | | | **295** | 6% | 11% | 15% | 11% | 2% | 7% | 9% | 2% | 12% | |
|  | | | **132** | 100% | 100% | 100% | 100% | 100% | 100% | 100% | 100% | 100% | |
| **Control** | | | **150** | 100% | 100% | 100% | 100% | 100% | 100% | 100% | 100% | 100% | |
|  | | | **196** | 100% | 100% | 100% | 100% | 100% | 100% | 100% | 100% | 100% | |
|  | | | **235** | 100% | 100% | 100% | 100% | 100% | 100% | 100% | 100% | 100% | |
|  | | | **295** | 100% | 100% | 100% | 100% | 100% | 100% | 100% | 100% | 100% | |
