# Supplementary material for: Use of FFPE-derived DNA in next generation sequencing: DNA extraction methods
Source: PLoS One. 2019 Apr 11;14(4):e0211400. doi: 10.1371/journal.pone.0211400 (PMC6459541; doi:10.1371/journal.pone.0211400)
Supplement: S3 Table — (DOCX) [file pone.0211400.s006.docx]

**S3 Table :** Whole Exome Sequencing Metrics for the two library preparation methods and four selected DNA extraction methods

1. **Thruplex**

| **Tissue** | **Breast** | | | **Pancreas** | | | **Cerebellum** | | |
| --- | --- | --- | --- | --- | --- | --- | --- | --- | --- |
| **Methods*** | **MC** | **DR** | **IS** | **MC** | **DR** | **IS** | **MC** | **DR** | **IS** |
| **QGR-M** | 23 | 25.6 | 167 | 24 | 24.1 | 160 | 3 | 58.2 | 116 |
| **QA-M** | 20 | 30.7 | 167 | 24 | 25.6 | 168 | 2 | 64.3 | 116 |
| **QGR-A** | 22 | 20.5 | 164 | 20 | 22.7 | 157 | 2 | 62 | 114 |
| **TKM-A** | 19 | 27.6 | 152 | 20 | 32.9 | 157 | 7 | 59.2 | 122 |

*MC : Median Coverage ; DR : Duplication Rate; IS : Median Insert Size

**B. Ultra II**

| **Tissue** | **Breast** | | | **Pancreas** | | | **Cerebellum** | | |
| --- | --- | --- | --- | --- | --- | --- | --- | --- | --- |
| **Methods** | **MC** | **DR** | **IS** | **MC** | **DR** | **IS** | **MC** | **DR** | **IS** |
| **QGR-M** | 26 | 9.7 | 159 | 25 | 9.6 | 157 | 16 | 34.6 | 125 |
| **QA-M** | 30 | 16.3 | 182 | 29 | 12.4 | 172 | 6 | 37.8 | 127 |
| **QGR-A** | 28 | 13.1 | 170 | 25 | 14.9 | 174 | 5 | 30.7 | 124 |
| **TKM-A** | 25 | 11 | 160 | 19 | 8.2 | 157 | 18 | 38.3 | 146 |

*MC : Median Coverage ; DR : Duplication Rate; IS : Median Insert Size
