# Supplementary material for: Use of FFPE-derived DNA in next generation sequencing: DNA extraction methods
Source: PLoS One. 2019 Apr 11;14(4):e0211400. doi: 10.1371/journal.pone.0211400 (PMC6459541; doi:10.1371/journal.pone.0211400)
Supplement: S4 Table — (DOCX) [file pone.0211400.s007.docx]

**S4 Table Coverage and Insert Size Metrics for the two panels**

1. **Comprehensive Cancer Panel**

| **Tissue** | **Breast** | | **Pancreas** | | **Cerebellum** | |
| --- | --- | --- | --- | --- | --- | --- |
| **Methods** | MC | IS | MC | IS | MC | IS |
| **QGR-M** | 833.96 | 149 | 1001.37 | 151 | 117.57 | 97 |
| **QA-M** | 583.73 | 156 | 1090.85 | 150 | 124.46 | 93 |
| **QGR-A** | 939.32 | 148 | 1104.83 | 148 | 157.17 | 96 |
| **TKM-A** | 956.33 | 152 | 1140.07 | 152 | 366.34 | 114 |

*MC : Median Coverage ;IS : Insert Size

**B. Breast Cancer Panel**

| **Tissue** | **Breast** | |
| --- | --- | --- |
| **Methods** | MC | IS |
| **QGR-M** | 749.12 | 91 |
| **QA-M** | 1089.31 | 124 |
| **QGR-A** | 731.02 | 115 |
| **TKM-A** | 1609.23 | 125 |

*MC : Median Coverage ; IS : Insert Size
